# Development of a novel cellular model of Alzheimer’s disease utilizing neurosphere cultures derived from B6C3-Tg(*APPswe,PSEN1dE9*)85Dbo/J embryonic mouse brain

**DOI:** 10.1186/2193-1801-3-161

**Published:** 2014-03-26

**Authors:** Pankaj S Ghate, Himakshi Sidhar, George A Carlson, Ranjit K Giri

**Affiliations:** National Brain Research Centre, Manesar, Haryana India; McLaughlin Research Institute, Great Falls, MT USA; Molecular and Cellular Neuroscience Division, National Brain Research Centre, Manesar, Haryana 122051 India

**Keywords:** Neurosphere, Transgenic, APPswe, PSEN1dE9, Cellular model, Alzheimer’s disease, Amyloid-β

## Abstract

Increased production, oligomerization and aggregation of amyloid-β (Aβ) peptides are hallmark pathologies of Alzheimer’s disease (AD). Expressing familial AD mutations (amyloid precursor protein and/or presenilins mutations), the Aβ-pathologies of AD has been recapitulated in animal models of AD. Very few primary cell culture-based models of AD are available and they exhibit very weak Aβ-pathologies compared to what is seen in AD patients and animal models of AD. CNS stem/progenitor cells are present in both embryonic and adult brains. They can be isolated, grown as neurospheres and differentiated into neurons, astrocytes and oligodendrocytes. It is not yet known whether CNS stem/progenitor cells can support the production of Aβ peptides in culture. In this report, we have established Aβ-pathologies such as production, secretion, oligomerization and aggregation of Aβ peptides utilizing neurosphere cultures to create a new cellular model of AD. These cultures were developed from E15 embryonic brains of transgenic mice carrying the Swedish mutations in humanized mouse APP cDNA and the exon-9 deleted human presenilin 1 cDNA both regulated by mouse prion protein gene (*Prnp*) promoter. Results demonstrated the expression of transgene transcripts, APPswe protein and its processed products only in transgene positive neurosphere cultures. These cultures generate and secrete both Aβ40 and Aβ42 peptides into culture medium at levels comparable to the Aβ load in the brain of AD patients and animal models of AD, and produce pathogenic oligomers of Aβ peptides. The Aβ42/Aβ40 ratio in the medium of transgene positive neurosphere cultures is higher than any known cellular models of AD. Conformation dependent immunocytochemistry demonstrated the possible presence of intracellular and extracellular aggregation of Aβ peptides in neurosphere cultures, which are also seen in AD brain and animal models of AD. Collectively, our neurosphere cultures provide robust Aβ-pathologies of AD better than existing cellular model of Alzheimer’s disease.

**Electronic supplementary material:**

The online version of this article (doi:10.1186/2193-1801-3-161) contains supplementary material, which is available to authorized users.

## Introduction

Alzheimer’s disease (AD) is a chronic, irreversible and progressive neurodegenerative disease. AD is characterized by extracellular deposition of amyloid-β (Aβ) peptides as senile plaques (Glenner and Wong 1984), intraneuronal neurofibrillary tangles (NFT) (Kosik et al. 1986; Wood et al. 1986), astrogliosis (Rodriguez et al. 2009), microglial activation (Giri et al. 2003) and loss of synapses and neurons in the brain (Selkoe 2002; Whitehouse et al. 1982). Genetic linkage analysis of familial Alzheimer’s disease (FAD) identified amyloid precursor protein (*APP*) (Chartier-Harlin et al. 1991; Goate et al. 1991; St George-Hyslop et al. 1987) and presenilins i.e. *PSEN1* (Citron et al. 1997; Sherrington et al. 1995) and *PSEN2* (Levy-Lahad et al. 1995) as the causative genes in FAD. Mutations in these genes are linked to increased Aβ formation, specifically the more fibrillogenic Aβ42 peptides (Borchelt et al. 1996b; Scheuner et al. 1996; Tomita et al. 1997), which led to the formulation of the amyloid-β cascade hypothesis. The amyloid-β cascade hypothesis states that Aβ production is the earliest event in the cascade that eventually leads to AD associated neurodegeneration (Hardy and Allsop 1991; Sommer 2002). Utilizing this hypothesis, transgenic animals were created using human genes linked to FAD such as *APP* and *PSEN1*. These transgenic animals show increased production and deposition of Aβ peptides (senile plaques) and memory impairments. However, the majority of such AD animal models take upto a year to develop signs of disease in the brain (Eimer and Vassar 2013; Youmans et al. 2012). Therefore, quicker, cheaper and reproducible alternative models were explored utilizing cell culture based systems.

Various cancerous cell lines (Wang et al. 2000) and primary cortical neurons (LeBlanc 1995; Lorenzo and Yankner 1994; Takashima et al. 1993; Yankner et al. 1990) have been extensively utilized to study the toxic effect of Aβ peptides. Cancerous cell lines with neuronal origin (Borchelt et al. 1996b; Cai et al. 1993) and non-neuronal origin (Citron et al. 1997; Haass et al. 1992) that express FAD genes produce Aβ peptides and are useful for the study of Aβ genesis, stability and intracellular trafficking (Vassar et al. 1999; Wertkin et al. 1993). However, these models are limited by their genetic instability over multiple passages, their rapid growth rate and their inability to model mature brain cell types. Primary cell cultures expressing FAD genes may model AD better *in vitro*, and thus warrant investigation. Primary hippocampal cell cultures from transgenic animal models for AD express Aβ peptides endogenously (Trinchese et al. 2004; Yun et al. 2007). However, these cultures are difficult to maintain for more than 3 weeks (Brewer and Torricelli 2007) and cannot be passaged. Therefore, long-term experimentation and need for repetition demands frequent tissue harvests when using this system. Similarly, slice cultures cannot be maintained for more than few weeks (De Simoni and Yu 2006). It has been well documented that, CNS stem/progenitor cells are present in both embryonic and adult brain. These cells can be isolated and grown as neurospheres in substrate free vessels over several passages like transformed cell lines and maintain the properties of stem/progenitor cells (Brustle et al. 1997; Ray and Gage 2006; Reynolds and Weiss 1992; Uchida et al. 2000). Most importantly, neurosphere cultures can be differentiated into major cell types of an adult brain such as neurons, astrocytes and oligodendrocytes (Gritti et al. 1996; Ray and Gage 2006). Although neural stem cells have been utilized to study the effect of Aβ peptides, to our knowledge, there is no report indicating the use of CNS stem/progenitor cells to model beta amyloid pathology of AD *in vitro*.

In the present report, we have combined the transgenic and CNS stem/progenitor cell culture technologies to develop a novel platform to model the pathological processing of mutant human APPswe protein for Aβ genesis, oligomerization and aggregation, the initial events of AD pathogenesis. Neurosphere cultures were established from AD transgenic (*APPswe,PSEN1dE9*) mice embryos. Neurosphere cultures positive for transgenes (Tg+ve) express both transgenes at the mRNA level and express humanized APP and its proteolytic products including Aβ peptides. Analysis of Tg+ve neurosphere lysates demonstrated the presence of both monomeric and various oligomeric Aβ peptides similar to an 18-month old Tg+ve mouse brain homogenate. Tg+ve neurosphere cultures secrete a large amount of human Aβ peptides that consist of Aβ40 and Aβ42 with a very high Aβ42/Aβ40 ratio comparable to that of human AD brain homogenates and more than any cellular model of AD. Tg+ve culture supernatants also contain monomeric and various pathogenic Aβ peptide oligomers (ranging from 2-mer to 12-mer; the Aβ star oligomer). In addition, conformation-dependent immunocytochemistry demonstrated the presence of intracellular and extracellular Aβ peptides within neurospheres. Thus, our results provide compelling evidence for Aβ peptide genesis, secretion, oligomerization and aggregation in Tg+ve neurosphere cultures better than any existing cellular model of AD.

## Materials and methods

### Ethics statement

All experiments on animals were conducted in accordance with guidelines approved by the committee for the purpose of control and supervision of experiments on animals (Regd. No. 464/a/CPCSEA). All animal procedures were reviewed and approved by National Brain Research Centre (NBRC) animal ethics committee (NBRC/IAEC/2008/44).

### Mice

B6C3-Tg(*APPswe,PSEN1dE9*)85Dbo/J mice were obtained from the Jackson Laboratory (Bar Harbor, Maine, USA) and maintained as a mouse line in NBRC. Tg+ve mice express APPswe (K670N and M671L) mutations in humanized mouse APP cDNA and exon 9-deleted human presenilin 1 (PSEN1dE9) cDNA under the control of the mouse prion protein (*Prnp*) gene promoter. Both these transgenes are integrated at a same locus resulting 50% of litters are hemizygous for both APPswe and PSEN1dE9 transgenes and rest 50% as wild type controls from a cross between hemizygous transgenics to wild type (Borchelt et al. 1996a; Jankowsky et al. 2004).

### Antibodies

Beta amyloid 1-16 monoclonal antibody (6E10), which is specific for human APP and some of its proteolytic products including Aβ peptides and anti-Aβ42 antibody (BA3-9) specific for human Aβ42 were purchased from Covance. Anti-nestin antibody was purchased from Chemicon. Anti-GAPDH, anti-β-tubulin III and anti-glial fibrillary acidic protein (GFAP) antibodies were purchased from Santacruz, Sigma-Aldrich and DAKO respectively. Secondary antibodies conjugated with HRP and Alexa-fluorophores were purchased from Pierce and Invitrogen respectively.

### Isolation of DNA and genotyping

Total genomic DNA was isolated from the tail of mouse during weaning using QIAamp DNA Mini kit (Qiagen). Genomic DNA (1 μl) was used to amplify hu*APPswe* and hu*PSEN1dE9* transgenes by polymerase chain reaction (PCR). Primers for APP and PSEN1 transgenes were purchased from Sigma. Primers sequences were obtained from the Jackson Laboratory. PCR products were resolved on 2% agarose gel and digital images of ethidium bromide stained gels were captured using ChemiDoc XRS^+^ gel doc system (BIO-RAD, USA).

### Neurosphere isolation

Embryos from a wild type female mouse bred with a hemizygous Tg(*APPswe,PSEN1dE9*) male mouse were harvested on embryonic date 15 (E15) and neurosphere (NS) cultures were isolated using earlier protocol (Giri et al. 2006). Briefly, whole brain was isolated from each embryo and triturated in 1 ml of neurobasal media supplemented with glutamax and antibiotics (all from Invitrogen, USA) using filtered 200 μl tips to obtain homogeneous cell suspension. Each cell suspension was diluted further to 10 ml using same media and filtered through 45 μm mesh (Falcon, USA). The filtrates were centrifuged at 1000 rpm (~100×g) for 5 min at room temperature (RT). Cell pellets were gently triturated through filtered 200 μl tips and cultured in T75 non-adherent culture flask (Nunc, USA) in 15 ml of complete neurobasal media (neurobasal media supplemented with N2 supplement (1X), 2 mM Glutamax, Penicillin-streptomycin mix (1X), 20 ng/ml of recombinant huEGF, 10 ng/ml of recombinant huFGF-b (all from Invitrogen, USA) and 10 ng/ml of recombinant mouse LIF (Chemicon, USA). After 2-3 days in culture, distinct cell clumps were seen, collected and recultured in complete neurobasal media. After every 3-4 days, 50% of media was replaced with fresh complete neurobasal media. Usually, distinct spheres of cells are visible by 4-7 days of culture. These cultures took approximately one month of time to grow sufficiently to warrant further passage. Neurospheres were split at 1:3 ratios by triturating NS pellets. It is important to stress here that our culture system favors slower growth than others but was developed to allow infection by RML scrapie prions in NS cultures (Giri et al. 2006). Neurosphere culture conditions that favor faster growth are not infected with prions (Herva et al. 2010). Although our cultures grow slowly, they are highly enriched with nestin positive cells (CNS stem/progenitor cells) and generate neurons and astrocytes after differentiation (data not shown). Therefore, in this report, we have employed slow growing NS culture protocol to model Aβ genesis, oligomerization and aggregation *in vitro*. Genomic DNA from each NS line and mother’s tail were isolated and genotyped for transgenes as mentioned above. Nine NS lines have been established out of which, NS1-4 are used extensively in this report. Furthermore, two additional Tg-ve NS lines (NS6 and 7) were also used.

### Isolation of RNA

Total RNA was isolated from each NS culture using Trizol reagents (Invitrogen). Twenty microgram (μg) of total RNA was treated with amplification grade and RNase free DNase I (Invitrogen) as per manufacturer’s protocol. Concentration of RNA was measured using NanoVue Plus spectrophotometer (GE).

### RT-PCR

Twenty-five nanogram of DNase I treated total RNA from each NS line was reverse transcribed for cDNA preparation. HuAPPswe, huPSEN1dE9 and GAPDH transcripts were amplified by one-step reverse transcriptase PCR (RT-PCR) employing manufacturer’s protocol (Qiagen). The following primers were used for RT-PCR: APPswe, forward: 5′-TTCCCGTGAATGGAGAGAGTTC-3′; reverse: 5′-ATGAACTTCATATCCTGAGTCATGTCG-3′, PSEN1dE9, forward: 5′-GGTCCACTTCGTATGCTGGT-3′; reverse: 5′-TTCCCATTCCTCACTGAACC-3′. Primers for GAPDH were similar to that reported earlier (Usenko et al. 2009). PCR products were resolved using 2% agarose gel electrophoresis and digital images of ethidium bromide stained DNA fragments were captured in ChemiDoc XRS^+^ gel doc system.

### Detection of huAPPswe protein in neurosphere lysates

Neurospheres were harvested at the end of each passage by centrifuging the culture at 1000 rpm for 5 minutes at room temperature. Neurosphere pellets were lysed in RIPA buffer (150 mM NaCl; 10 mM Tris, pH 7.4; 0.5% Triton-X 100; 0.5% sodium deoxycholate; 0.1% SDS and 1X protease inhibitor cocktail). Protein concentration was determined by micro BCA protein assay kit (Pierce). Sixty μg of total protein was size fractionated in 15% Tris-Glycine polyacrylamide gel along with brain homogenates from 18-months old Tg+ve and Tg-ve control mice. Aβ42 was used as an additional positive control. Proteins were then transferred onto 0.2 μm PVDF membranes. Blots were incubated for 10 min in boiling phosphate buffered saline (PBS) bath for epitope retrieval (Ida et al. 1996; Swerdlow et al. 1986) followed by immunoblotting with 1000-fold diluted 6E10 antibody. The secondary antibody, anti-mouse IgG conjugated with HRP was used to visualize the bands and by using supersignal west pico chemiluminescent kit (Pierce) on X-ray film (Amersham). Time-lapse digital images were also captured using ChemiDoc XRS^+^ gel doc system. Digital images without saturation were used for densitometric and molecular weight analysis with ImageLab (version 3.0) software.

### Detection of Aβ peptides in culture media

Culture media from Tg-ve and Tg+ve neurosphere lines were collected at the end of each passage and after 4 days of media change. Culture media were centrifuged at 100 × g for 5 minutes at RT to sediment neurospheres, small cell clumps and cell debris. Twenty-three milliliters (ml) of culture medium were spin filtered in two batches in an Amicon ultra 15 (or 8 ml of medium in Amicon ultra 4) centrifugal filter (Millipore), which retain all proteins above 3 kDa using manufacturer’s protocol. Retentates were collected, aliquoted and stored at -80°C. Protein concentration, western blotting and immunoblotting with 6E10 antibody were perfomed as described earlier. To differentiate Aβ40 and Aβ42 peptides in the Aβ pool of culture supernatant, 60 μg of concentrated culture supernatants along with 5 ng of each Aβ40 and Aβ42 peptides (as positive controls) were size fractionated in 10% Bicine-Tris-Urea-polyacrylamide gel as described earlier (Wiltfang et al. 1997). Western blotting and detection of Aβ peptides was performed using 6E10 antibody. Imaging and densitometric analysis were performed as described above.

### Immunocytochemistry

Neurospheres were triturated to obtain single cells or small cell clumps preparation. Approximately, 100000 cells were seeded onto poly-D-lysine (PDL) coated 24-well cover glass plates (Greiner) or onto PDL coated precleaned 12 mm diameter glass coverslips. Cells were grown in complete medium for 3 days with a medium change after 1 day of seeding. After removing the medium and three PBS washes, cells were fixed in 4% formaldehyde (PFA) for 30 minutes at RT. Cells were washed thrice with PBS for 5 min each followed by permeabilization with 0.3% Triton-X 100 in PBS for 5 minutes at RT. Cells were blocked with 10% normal goat serum in washing buffer (0.1% BSA, 0.05% NaN_3_ in 1X PBS) for 1 hour at RT followed by overnight incubation with primary antibodies at 4°C. Cells were then washed five times (5 minutes each) followed by incubation with appropriate secondary antibodies conjugated with either Alexa 488 or Alexa 594 fluorophores for 1 hour at RT. After series of washes, cells were mounted in prolong gold anti-fade reagent containing DAPI (Molecular Probes).

### Conformation dependent immunocytochemistry (CDIC)

Under physiological conditions, Aβ peptides undergo conformational changes to form β-sheet, which are not efficiently reactive with 6E10 antibody (Rosen et al. 2010). Upon denaturation by formic acid (FA), the epitope of Aβ peptides of various oligomers and conformers unfolds and binds efficiently with 6E10 antibody. Such mechanisms were exploited to detect the possible presence of Aβ peptides within and outside the cells in neurospheres. In neurosphere monolayer cultures, cells were fixed and permeabilized. One group of NS cultures from each line was treated with 70% FA for 1 hour at RT and the other group left untreated. Immunostaining was performed using 6E10 antibody as described earlier. To detect intracellular and extracellular Aβ peptides within neurospheres, fixation of neurospheres was performed in 4% PFA overnight followed by serial passaging through 10%, 20% and 30% sucrose in PBS. Neurospheres were embedded in cryomedium and frozen in an ethanol-dry ice bath. Ten μm thick frozen NS sections were obtained using a cryotome (Leica). Immunodetection of Aβ peptides with or without 70% FA treatment was similar to that described above. In addition to 6E10, an antibody specific for Aβ42 peptide was used to detect Aβ42 peptides in NS sections exposed or not exposed to formic acid.

### Image acquisition and analysis

Images of all the samples within an experiment were acquired during the same session by using identical image acquisition settings for each fluorophore. For epi-fluorescence imaging, digital images were acquired at best focal plane using 40X planfluor objective lens and Axiocam HR RGB camera in a ZEISS Axiovert 200 M microscope supported by AxiovisionRel (version 4.6.3.0) software. Densitometric analysis of images was performed using ImageJ 1.42q software (NIH). Regions with same area were drawn around the cell body of individual non-overlapping cells and mean fluorescence intensity (gray) value for individual cell was measured. After background subtraction, mean gray values were compared between transgene negative and positive groups. For confocal imaging of neurosphere sections, 63X oil, 12 bits multi-stack (at 0.5 μm interval) images were acquired by LSM 510 confocal microscope (Zeiss). Maximum intensity projection of central three sections was made and analyzed. For analysis, a fix sized region tool was made and mean gray values from 25 non-overlapping regions within the image containing cell mass were obtained. Same region tool was used on images captured from one experiment in one sitting. Areas without cell mass were excluded. This approach was adopted as some neurospheres had more hollow space than others.

### Statistical analysis

Statistical analysis was performed by one-way ANOVA when data passed normality and equal variance test using Sigmastat 3.5 software. When data failed the above test, the groups were compared by non-parametric Kruskal-Wallis one-way ANOVA on ranks. In addition, non-parametric Dunn’s method was employed to analyze pair-wise multiple comparisons. *T*-test was also calculated using Excel (Microsoft). p-value ≤ 0.05 is considered statistically significant.

## Results

### Development of CNS stem/progenitor cells (neurosphere) cultures from B6C3-Tg(*APPswe*,*PSEN1dE9*)85Dbo/J mice

**Figure 1:**
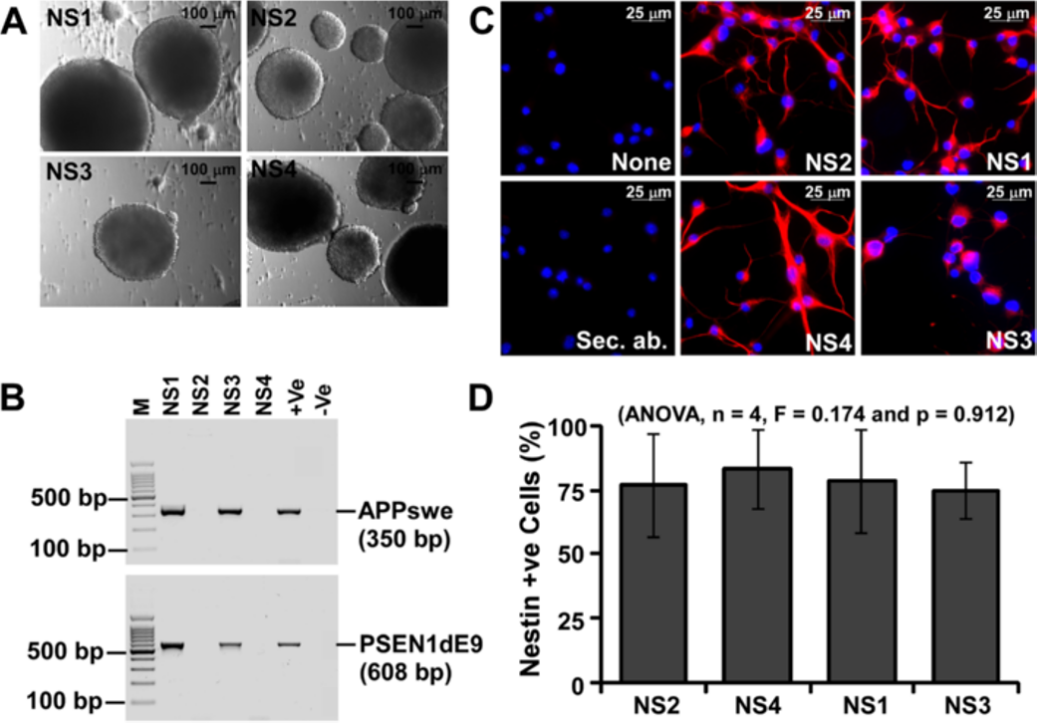
**Development of neurosphere (NS) cultures from B6C3-Tg(** ***APPswe,PSEN1dE9*** **)85Dbo/J E15 embryos. (A)** Cells from four mouse embryos (E15) were isolated and grown in non-treated tissue culture flasks to form neurospheres (NS1-NS4). **(B)** Genotyping of NS1-NS4 for hu*APPswe* (350-bp) and hu*PSEN1dE9* (608-bp) transgenes by PCR. **(C)** Expression of nestin in NS1-4 lines by immunofluorocytochemistry. **(D)** Cell type analysis of nestin expression in NS1-NS4 lines is represented as histograms of mean ± standard deviation (ANOVA, n = 4, F = 0.174, p = 0.912).

**Figure 2:**
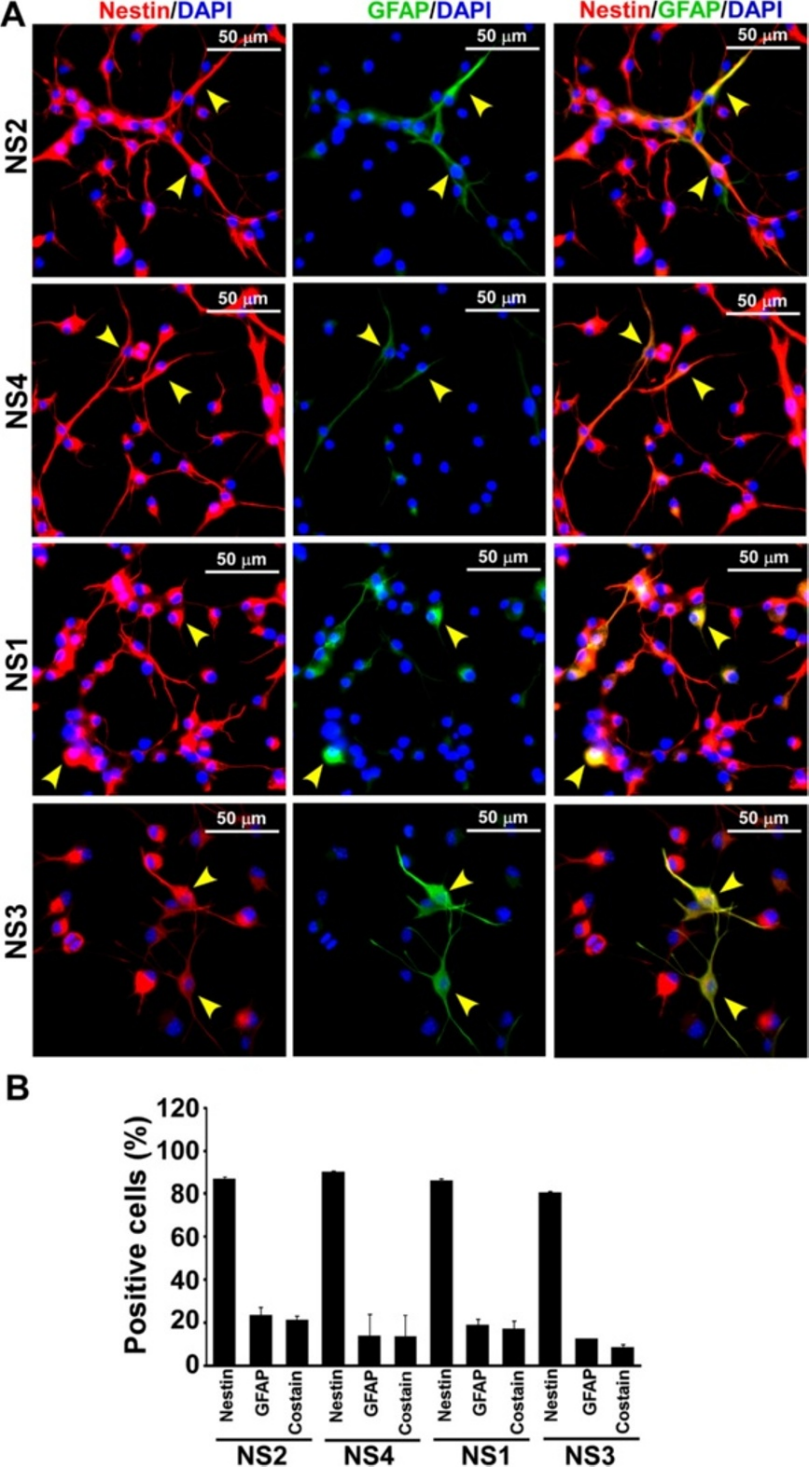
**Expression of nestin and glial fibrillary acidic protein (GFAP) in neurosphere cultures. (A)** Fixed monolayer culture of neurosphere cultures, cells were immunostained with anti-nestin and anti-GFAP antibodies (for details see materials and methods). All the images are displayed at the same intensity scale. Images show some nestin positive cells are also positive for GFAP (yellow arrowhead). **(B)** Cell type analysis was performed by counting the cells positive for each marker manually, using ImageJ 1.42q software (NIH). Percent cells positive either for nestin, GFAP or for both markers (co-stain) was plotted as histograms of mean + standard deviation of three independent experiments. No significant difference is found between GFAP stained cell populations among NS1, NS2, NS3 and NS4 lines regardless of the presence of transgenes.

**Figure 3:**
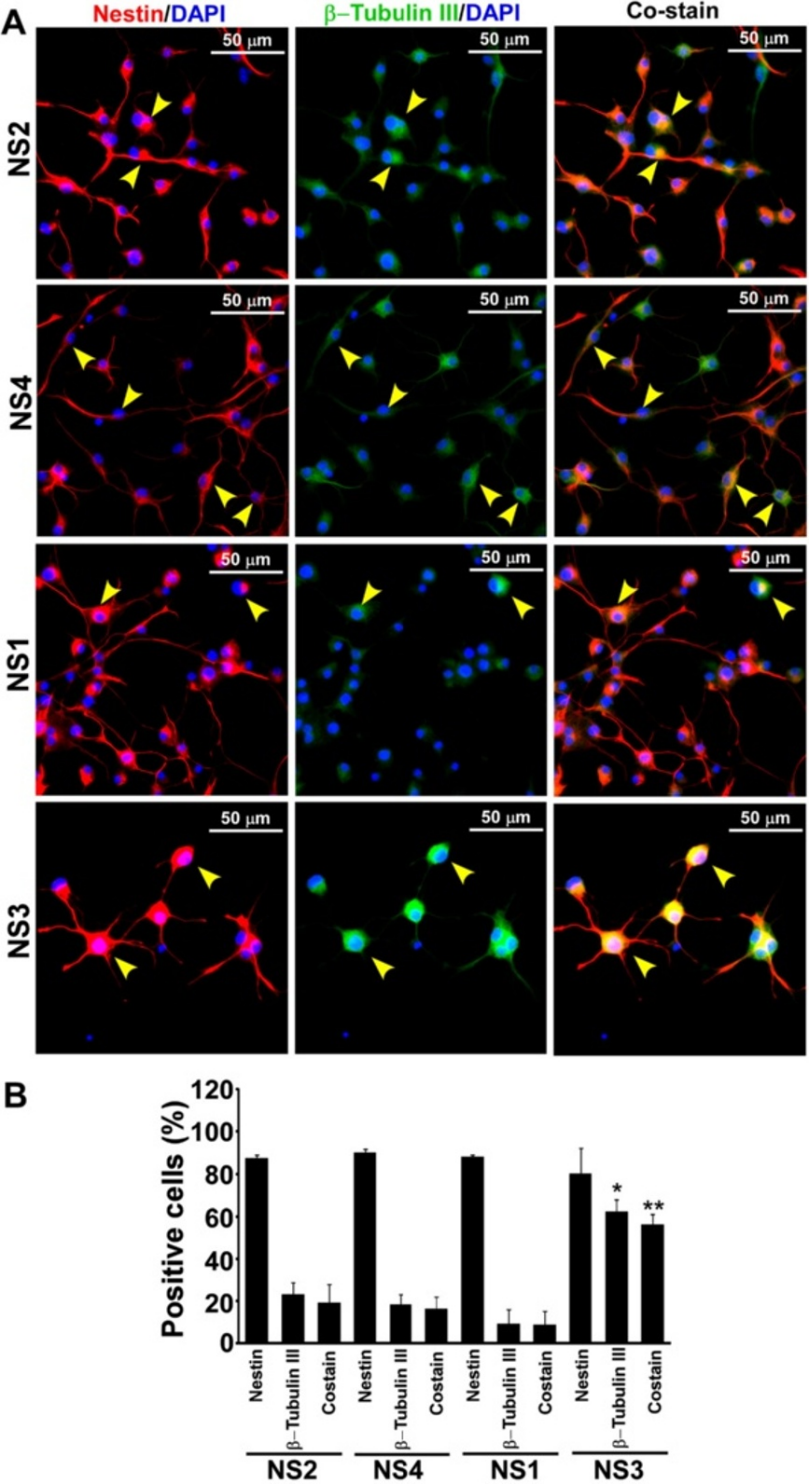
**Expression of nestin and β-tubulin III in NS cultures. (A)** Monolayer of cells was cultured as described earlier. Immunostaining with nestin and β-tubulin III antibodies, image acquisition, analysis and display are similar to Figure 2. Images demonstrate that some cells are positive for both nestin and β-tubulin III (yellow arrowheads). **(B)** Percent cells positive either for nestin, β-tubulin III or for both (co-stain) was plotted as histograms of mean + standard deviation from 6 images from two independent data sets. Significant increase in nestin and β-tubulin III co-stain cells is observed in NS3 line. *indicates p ≤ 0.05 when compared with other neurospheres and **indicates p ≤ 0.05 when compared with NS1 co-stain cells.

Embryonic day 15 (E15) brain cells isolated from B6C3-Tg(*APPswe*,*PSEN1dE9*)85Dbo/J mice grew as balls of cells termed as neurospheres (NS) within 4-5 days in culture (Figure 1A). PCR assay for hu*APPswe* and hu*PSEN1dE9* transgenes demonstrated the amplification of a 350-bp and a 608-bp DNA fragments respectively from NS1, NS3 and a Tg+ve mouse genomic DNA but not from NS2 and NS4 lines (Figure 1B) showing that, NS1 and NS3 lines are Tg+ve, whereas NS2 and NS4 are Tg-ve. Similar results were obtained at passages 0, 5 and 12 (data not shown). Neurosphere lines 5-9 were also genotyped. NS5, 8 and 9 were Tg+ve and NS6 and 7 were Tg-ve (data not shown). Immunocytochemical analysis of nestin on adherent cells from NS1-4 cultures indicated the expression of nestin in a majority of cells (Figure 1C). Cells without any antibody treatment (none) or treated with secondary antibody (Sec. ab.), show minimal immunosignal, indicating that both Tg-ve and Tg+ve NS lines express nestin, the most commonly used marker for CNS stem cells (Lendahl et al. 1990). Cell scoring analysis indicates that more than 75% of cells are positive for nestin in all the NS lines studied and no significant difference was observed between Tg+ve and Tg-ve lines (ANOVA, n = 4, F = 0.174, p = 0.912) (Figure 1D). When monolayer cultures from NS lines were co-immunostained with nestin and GFAP (a marker for glial cells), some cells are found to be co-stained for nestin and GFAP (Figure 2A). Cell type analysis in three independent experiments indicates approximately 21.6 ± 1.46, 13.6 ± 10.1, 17.3 ± 3.66 and 8.7 ± 1.1 percent of total cells were positive for both nestin and GFAP expression in NS2, NS4, NS1 and NS3 cultures respectively (Figure 2B). In addition, when cells were co-immunostained with nestin and β-tubulin III (a marker for young and mature neuron), approximately 19.3 ± 8.5, 16.4 ± 5.6, 8.7 ± 6.3 and 56.4 ± 4.6 percent of total cells are positive for both nestin and β-tubulin III (Figure 3A & B). Collectively, our NS cultures are enriched with CNS stem/progenitor cells.

### *APPswe*and *PSEN1dE9*transgene arrays are transcriptionally active and large numbers of cells express nestin and APP proteins in Tg+ve neurosphere lines

**Figure 4:**
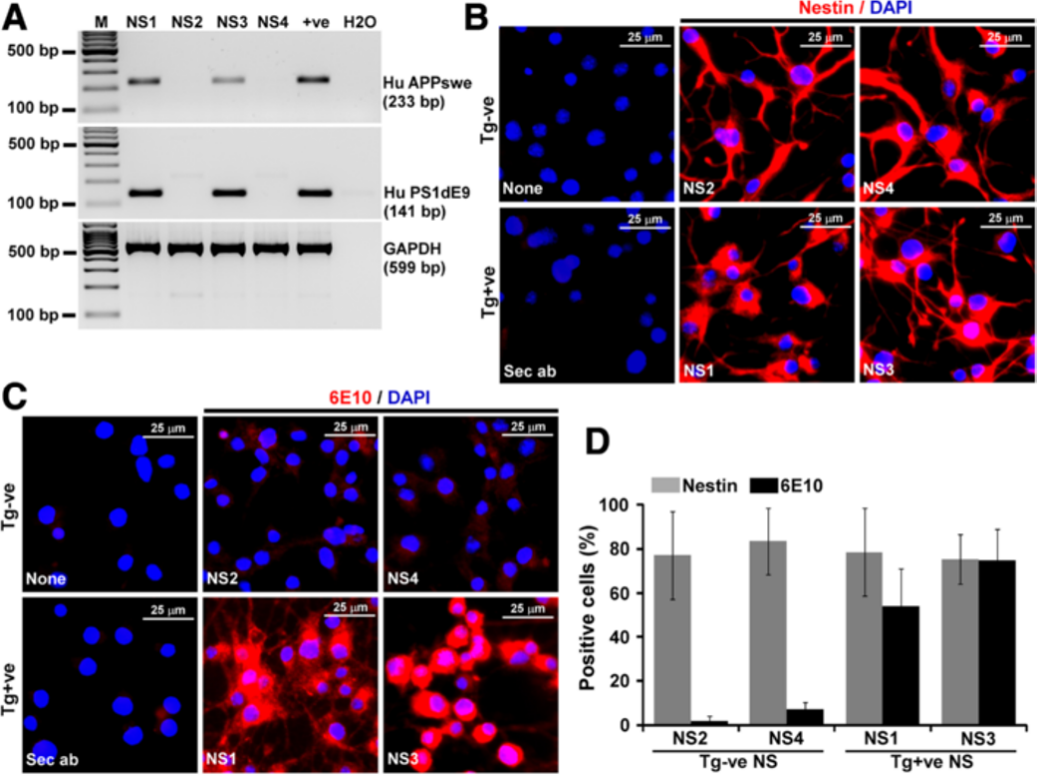
**Transgene positive neurosphere cultures transcribe both the transgenes, express humanized APP and co-express nestin. (A)** Detection of huAPPswe (233-bp) and huPSEN1dE9 (141-bp) transcripts in neurosphere lines by RT-PCR analysis. A 599-bp GAPDH fragment was amplified to validate equal RNA loading in all NS lines. **(B)** Immunofluorocytochemistry of nestin shows the majority of cells in all neurosphere lines express nestin. **(C)** Fluorescence images show expression of huAPPswe protein only in Tg+ve neurospheres using 6E10 antibody. Nuclei were stained with DAPI. **(D)** Fluorescence intensity from individual cells was derived and intensity above secondary antibody signal was considered as positive. Percentage of cells positive for each antibody from three independent experiments is demonstrated as histograms of mean ± standard deviation.

Reverse transcription and PCR analysis on DNase I treated RNA isolated from NS1-4 cultures demonstrated the amplification of 233-bp and 141-bp amplicons specific for huAPPswe and huPSEN1dE9 transcripts respectively in NS1 and NS3 lines but not in NS2 and NS4 lines (Figure 4A). GAPDH mRNA expression was similar in all NS cultures indicating the equal loading of RNA from all NS lines. An RT-minus control PCR on DNase I treated RNA from NS lines failed to amplify PSEN1dE9 (data not shown) suggesting the APPswe and PSEN1dE9 amplified products in NS1 and NS3 lines came from mRNA and not from DNA. To study nestin and APP co-expressing cells in Tg+ve NS cultures, we utilized immunofluorocytochemistry along with cell count analysis. Approximately, 77 ± 20, 83 ± 15, 78 ± 20 and 75 ± 11 percent of total cells analyzed from three independent experiments express nestin in NS2, NS4, NS1 and NS3 cultures respectively (Figure 4B & D). Immunostaining analysis (n = 3 independent experiments) with 6E10 antibody showed only 1.8 ± 2.2, 7 ± 3.3 in Tg-ve NS2 and NS4 lines respectively but 54 ± 17.12 and 75 ± 13.6 percent cells positive for APP expression in Tg+ve NS1 and NS3 cultures respectively (Figure 4C & D). Since nestin as well as APP expression is seen in more than 50% of total cells in Tg+ve NS cultures, it is postulated that nestin positive cells also express APP protein. Collectively, promoters for both the transgenes are active in neural stem cells and express APP protein in Tg+ve NS cultures, a prerequisite condition for its proteolytic processing towards Aβ peptide formation.

### Cells in Transgene positive neurosphere lines co-express human APP protein and β-tubulin III or GFAP

**Figure 5:**
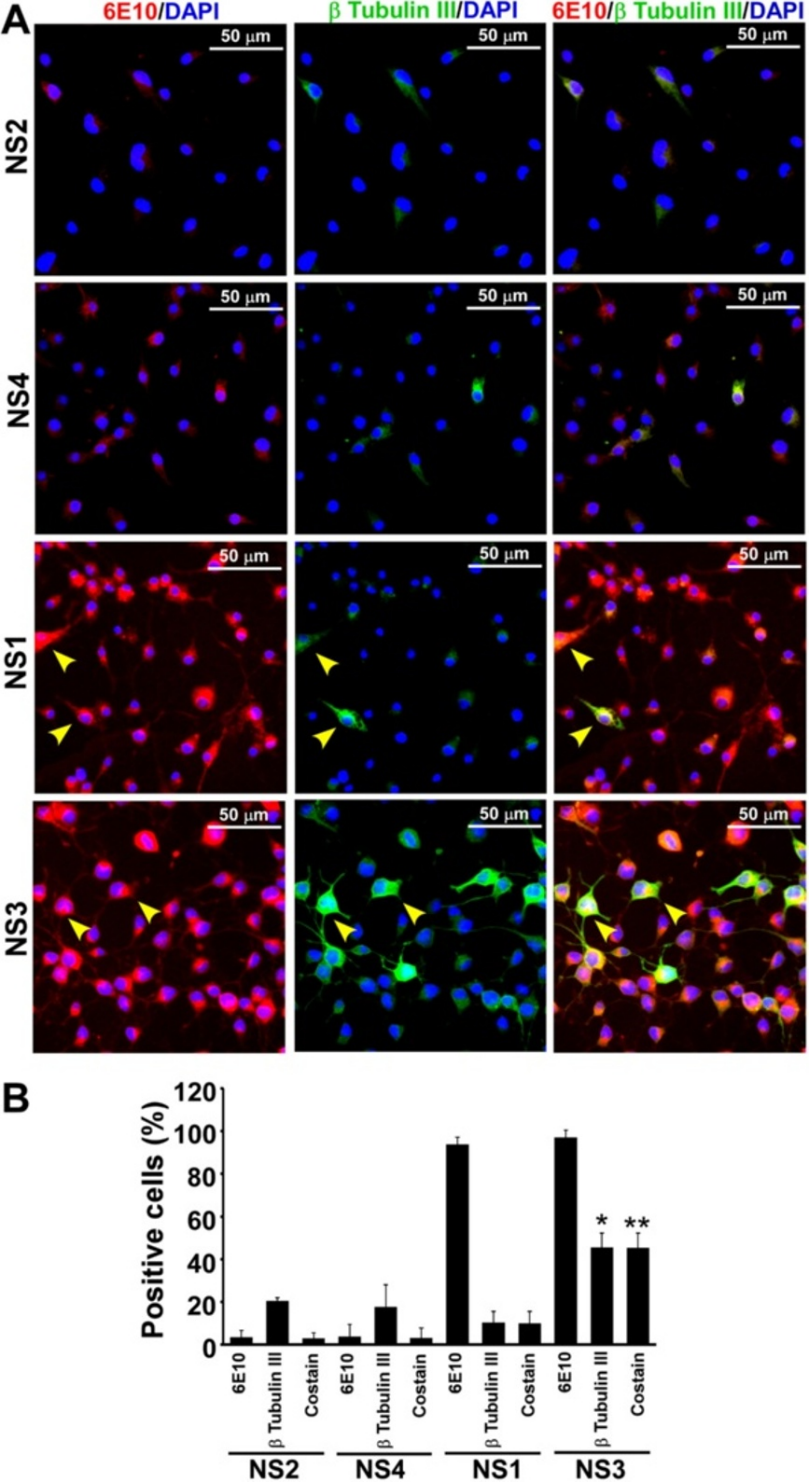
**Expression of APP and β-tubulin III proteins in neurosphere monolayer cultures. (A)** Triturated cells from NS cultures were grown on PDL coated optical cover-glass plates or glass coverslips for 3 days in complete media. Immunostaining with 6E10 (specific for human APP) and β-tubulin III antibodies, image acquisition, analysis and display are similar to Figure 2. Images demonstrate presence of cells positive for both APP and β-tubulin III in NS1 and NS3 (yellow arrowheads). **(B)** Percent cells positive either for APP or β-tubulin III or both (co-stain) are plotted as histograms of mean + standard deviation from three independent data sets. Significantly more APP and β-tubulin III co-stained cells are observed in NS3 line. *indicates p ≤ 0.05 when compared with other neurospheres and **indicates p ≤ 0.05 when compared with NS1 co-stained cells.

**Figure 6:**
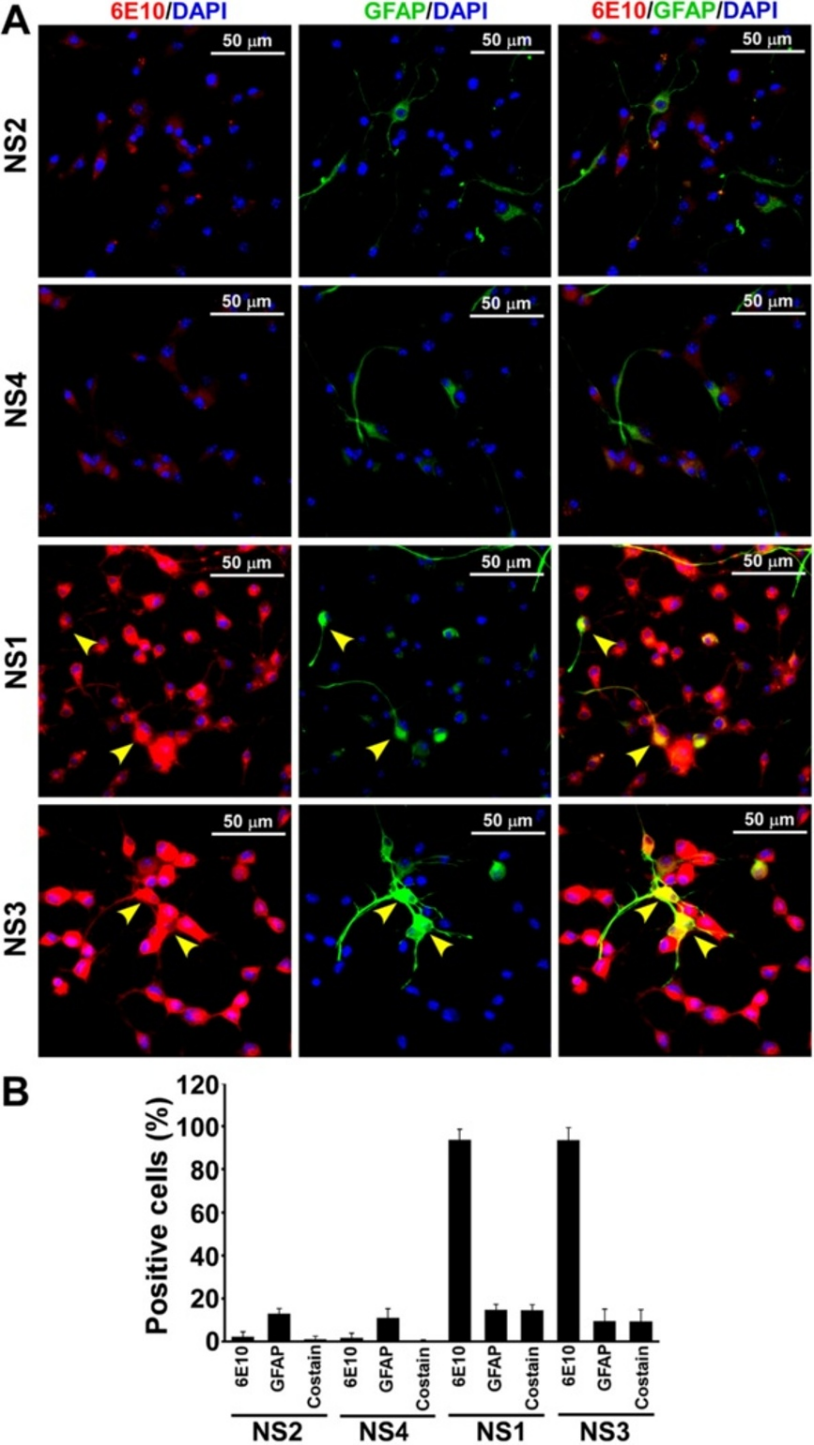
**Expression of APP and GFAP proteins in neurosphere monolayer cultures. (A)** Immunostaining of APP and GFAP on cell monolayers were performed as described earlier. Images demonstrate that some cells are positive for both APP and GFAP (yellow arrowheads) in Tg+ve NS lines. **(B)** Percent cells positive either for APP, GFAP or both (co-stain) are plotted as histograms of mean + standard deviation from four independent experiments.

Immunofluorocytochemical analysis of β-tubulin III expression demonstrates few cells are positive in Tg-ve NS2 and NS4 cultures and as expected, these cells are negative for APP expression. In Tg+ve NS1 and NS3 cultures, β-tubulin III positive cells co-localize with APP expression (Figure 5A). Cell type analysis of these cells from three independent experiments indicates almost all the β-tubulin III positive cells in NS1 (10.3 ± 5.3%) and NS3 (45.4 ± 6.8%) cultures also express APP protein (Figure 5B). When cells were co-immunostained with APP and GFAP, the results show 13.01 ± 2.3% and 11 ± 4.1% of total cells express GFAP in Tg-ve NS2 and NS4 cultures, and are not co-stained for APP expression (Figure 6B). In Tg+ve NS cultures, approximately, 14.8 ± 2.4% and 9.55 ± 5.5% cells are positive for GFAP expression in NS1 and NS3 respectively (Figure 6A & B). Cell type analysis shows that the majority of them also express APP (Figure 6B). Thus, β-tubulin III +ve neuronal and GFAP +ve astroglial progenitors are present in our NS cultures and express APP protein in Tg+ve neurosphere cultures.

### Transgene positive neurosphere lines express full-length APPswe protein and generate amyloid-β (Aβ) monomers and pathogenic oligomers

**Figure 7:**
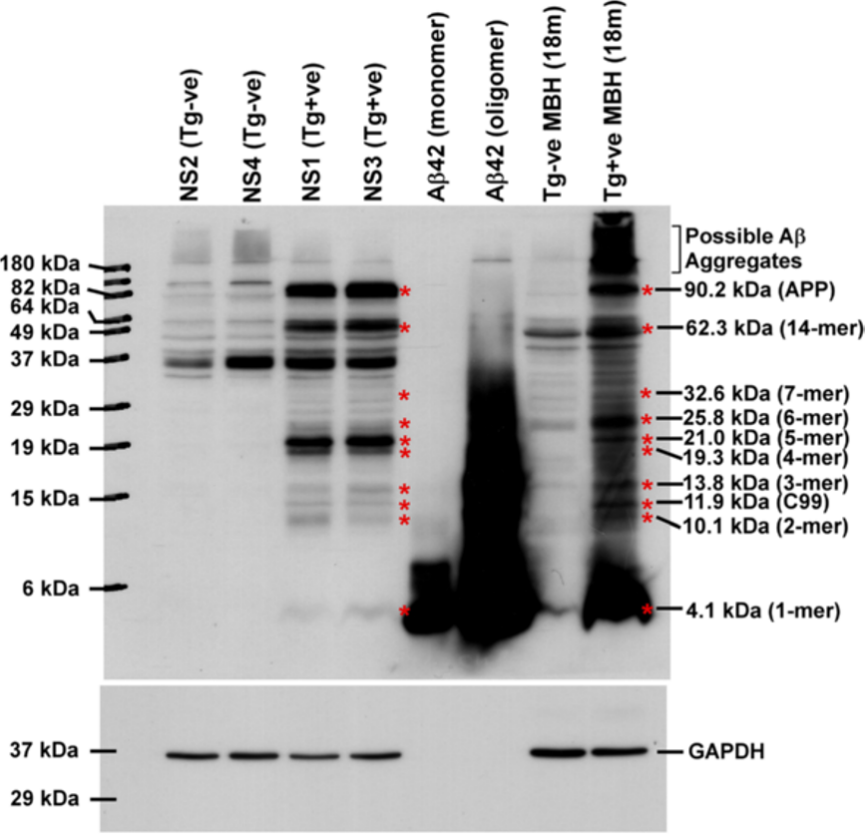
**Expression of huAPP and its proteolytic fragments in Tg+ve neurosphere lines.** Sixty microgram of total protein from each NS lysate was western blotted onto 0.2 μm PVDF membrane and immunoblotted with 6E10 antibody after HIER. Images clearly indicate the presence of huAPPswe full-length protein only in NS1, NS3 and 18 month (18 m) old Tg+ve mouse brain homogenate (MBH) but not in NS2, NS4 and 18 m old Tg-ve MBH. Monomeric and 2 days old oligomeric Aβ42 peptides were taken as positive controls. *indicates bands in common between Tg+ve neurosphere lysates and 18 m old Tg+ve MBH but not present in Tg-ve neurosphere lysates and 18 m old Tg-ve MBH. Molecular weight analysis indicates these bands could represent Aβ monomers to various oligomers as mentioned. Membranes were stripped and immunoblotted with GAPDH antibody to verify differences in protein loading. Data represents four independent experiments.

Western blot analysis of NS lysates from NS1-4 lines with 6E10 antibody clearly demonstrated the expression of full length huAPPswe (90.2 kDa) and its processed peptides in NS1, NS3 and Tg+ve mouse brain homogenate (positive control) but not in NS2, NS4 and Tg-ve mouse brain homogenate (negative control). A fragment close to 12 kDa was seen only in Tg+ve NS lines and in Tg+ve MBH, which likely represents the C99 fragment (APP β-carboxy-terminal fragment). Interestingly, additional bands ranging from 10.1 kDa to 62.3 kDa were also seen exclusively in Tg+ve NS lines and Tg+ve MBH. Molecular weight analysis of these bands corresponds to 2 to 14-mers of Aβ oligomers (Figure 7). In addition, monomeric Aβ peptides were detected only in Tg+ve but not in Tg-ve NS lysates. However, monomeric and oligomeric Aβ peptides intensities are weaker in Tg+ve NS lysates than in Tg+ve MBH. It is important to mention here that Tg+ve MBH contains both extracellular and intracellular Aβ peptides whereas NS lysates contain mostly intracellular Aβ peptides. Collectively, Tg+ve NS cultures express APP and its proteolytic peptides including Aβ peptides in monomeric and pathogenic oligomeric forms.

### Transgene positive neurosphere cultures secrete large amount of Aβ peptides into culture media

**Figure 8:**
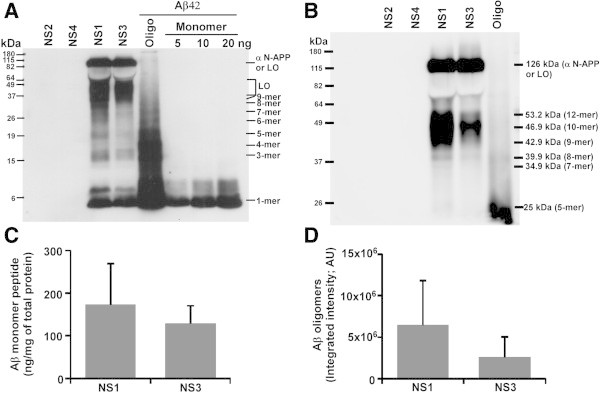
**Detection of human Aβ peptides in neurosphere culture supernatants. (A)** Sixty microgram of total protein from 80 to 100-fold concentrated neurosphere culture supernatant were size fractionated in Tris-Glycine-SDS-PAGE along with monomeric and oligomeric Aβ42 peptides as positive controls, transferred onto 0.2 μm PVDF membrane and immunoblotted with 6E10 antibody after heat induced epitope retrieval (HIER). Chemiluminescence digital images show the presence of Aβ monomers and various oligomeric Aβ peptides only in Tg+ve but not in Tg-ve neurosphere culture supernatants. Molecular weight analysis of these bands was compatible with the oligomers indicated. **(B)** A longer electrophoresis of the same samples resolved high molecular weight oligomers as potential Aβ 10-mers and 12-mers. (**C)** Densitometric analysis was used to measure the monomeric Aβ peptides in NS culture supernatants employing known amounts of Aβ42 as standards. **(D)** Densitometric analysis of Aβ oligomers (2-mer to 64 kDa) is presented as histograms of mean + standard deviation. Data represents four independent experiments. LO = Large Oligomer.

Since Aβ peptide levels were lower in Tg+ve NS lysates than Tg+ve MBH and Aβ peptides are deposited as senile plaques in the extracellular matrix of AD brain, we reasoned that most of the Aβ peptides might be secreted into culture media. Western blot analysis of concentrated NS culture supernatants demonstrated the presence of human Aβ peptides only from NS1 and NS3 cultures (Figure 8A). Oligomers (3-9-mer) and large oligomers of Aβ peptides are also seen in NS1 and NS3 culture supernatants (Figure 8A). A longer electrophoresis of the same samples in a separate polyacrylamide gel exhibited the presence of 10 and 12-mer Aβ oligomers in NS1 and NS3 culture supernatants (Figure 8B). Using known amount of Aβ42 peptides as standards, densitometric analysis of monomeric Aβ peptides in NS1 and NS3 culture supernatant was found to be 173 ± 96 ng and 128 ± 42 ng per milligram of total protein respectively (n = 4 independent experiments) (Figure 8C). In addition, the densitometric analysis of total oligomeric fractions (ranging from 2-mer to large oligomers) indicated NS1 culture supernatant has approximately 2.5-fold more Aβ oligomeric forms than NS3 culture supernatant (Figure 8D). Taken together, Tg+ve neurosphere cultures secrete both monomeric and wide range of Aβ oligomeric isoforms, a signature of AD pathology (Lesne et al. 2013).

**Figure 9:**
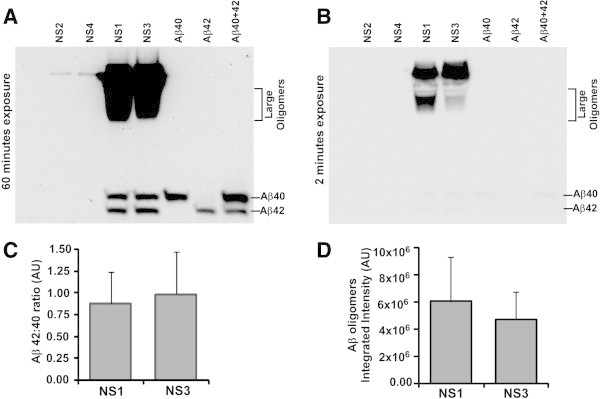
**Amyloid-β peptides in Tg+ve neurosphere culture supernatants contain Aβ40 and Aβ42 peptides. (A)** Sixty microgram of total protein from concentrated neurosphere culture supernatants were size fractionated in 10% Tris-Bicine-Urea-SDS-PAGE along with 5 ng of monomeric Aβ40 and Aβ42 peptides, followed by immunoblotting with 6E10 antibody after HIER. Tg+ve NS1 and NS3 neurosphere culture supernatants contain both Aβ40 and Aβ42 peptides but they are not seen in Tg-ve neurosphere culture supernatants. **(B)** Images from a shorter exposure show large oligomeric Aβ levels are higher in NS1 than NS3 culture supernatants. **(C)** Densitometric analysis of Aβ40 and Aβ42 peptide bands indicates the ratio of Aβ42/Aβ40 in NS1 and NS3 culture supernatants as 0.875 ± 0.336 and 0.989 ± 0.487 respectively. **(D)** Densitometric analysis of large Aβ oligomers is presented as histogram of mean + standard deviation from four separate experiments.

Next we wanted to resolve monomeric Aβ pool to identify various Aβ peptides secreted into the culture medium. Results showed the Aβ40 and Aβ42 peptides are only produced by Tg+ve (NS1 and NS3) but not by Tg-ve (NS2 and NS4) NS lines (Figure 9A). Densitometric analysis of Aβ40 and Aβ42 bands from four separate experiments indicated Aβ42/Aβ40 ratio as 0.875 ± 0.336 and 0.989 ± 0.487 for NS1 and NS3 respectively (Figure 9C). Similar to our previous result, NS1 culture supernatants contain approximately 2.2-fold more oligomeric Aβ peptides than NS3 culture supernatants (Figure 9B & D).

### Cells in transgene positive neurosphere lines have intracellular Aβ peptides

**Figure 10:**
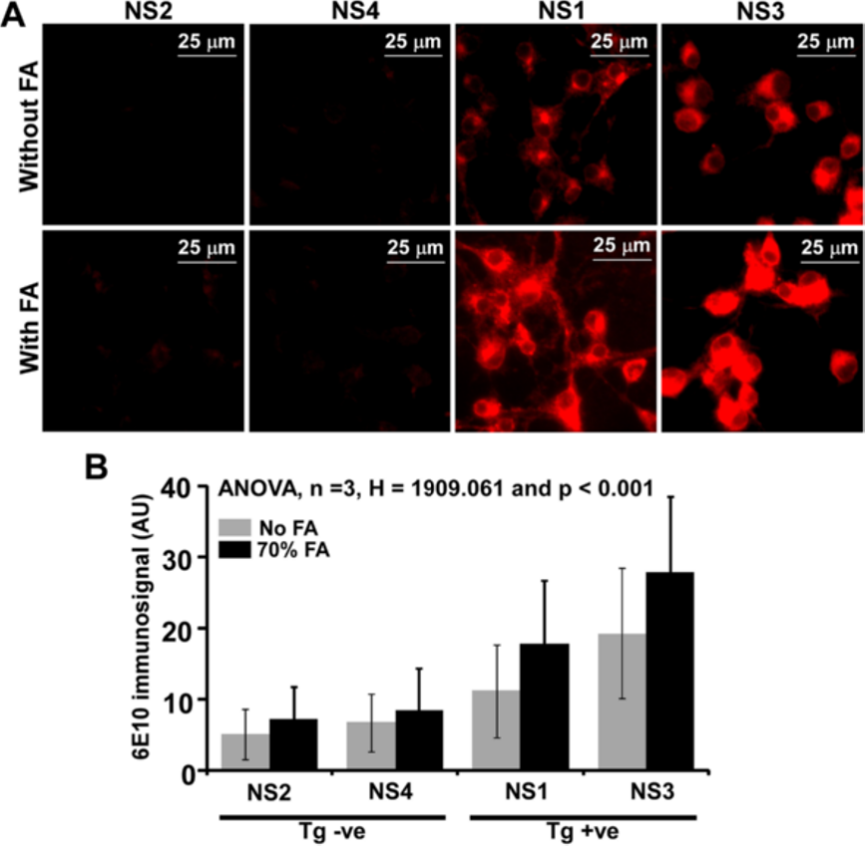
**Detection of intracellular Aβ peptides by CDIC. (A)** Fixed and permeabilized adherent cells from neurosphere cultures were either treated with formic acid (FA) or left untreated followed by immunostaining with 6E10 antibody. Detection was done using Alexa 594 labeled secondary antibody. **(B)** Quantification of fluorescence intensity indicates that Tg+ve NS lines show significant increased signal over Tg-ve lines in both the treatments. FA treatment significantly increased signal in Tg+ve NS lines (NS1 and NS3) compared to non-treated counterparts suggesting the possible presence of intracellular Aβ peptides (non-parametric one-way ANOVA, n = 3, H = 1909.061 and p < 0.001).

Western blot analysis of NS lysates indicated the presence of intracellular localization of Aβ peptides. To validate intracellular pool of Aβ peptides, we utilized conformation dependent interaction of Aβ peptides with 6E10 antibody. However, 6E10 antibody interacts with full length human APP as well as human Aβ peptides which requires epitope retrieval (Rosen et al. 2010). To dissect the effect of epitope retrieval on full-length APP and Aβ peptides by 6E10 antibody, we performed western blot analysis of Tg+ve mouse brain homogenate using 6E10 antibody with or without heat induced epitope retrieval (HIER) (Ida et al. 1996). The densitometric analysis of APP, Aβ and GAPDH bands from both the conditions indicates HIER induced increased Aβ signal is 20-fold higher than HIER induced increased APP signal and 12-fold higher than HIER induced increased GAPDH signal (Additional file 1: Figure S1). Thus, Aβ peptides require more epitope retrieval than full length APP for its interaction with 6E10 antibody. Similarly, formic acid (FA) treatment has been found to be essential for the detection of aggregated intraneuronal Aβ peptides in the brain section of a mouse model of AD (Christensen et al. 2009) suggesting Aβ epitopes are hidden within aggregated Aβ structures. Immunofluorescent staining of adherent cells from NS cultures demonstrated significant higher immunosignal towards 6E10 in Tg+ve than Tg-ve NS lines without FA treatment (Figure 10A, upper panel &10B). However, after FA treatment, Tg+ve cells showed dramatic increased immunosignal over untreated counterparts, whereas, as expected, Tg-ve cells showed marginal increase (Figure 10A, lower panel &10B). Thus, increased immunosignal in Tg+ve neurosphere cultures to epitope retrieval might represents intracellular monomeric and oligomeric Aβ peptides.

### Accumulation of extracellular and intracellular Aβ peptides within transgenic positive neurospheres

**Figure 11:**
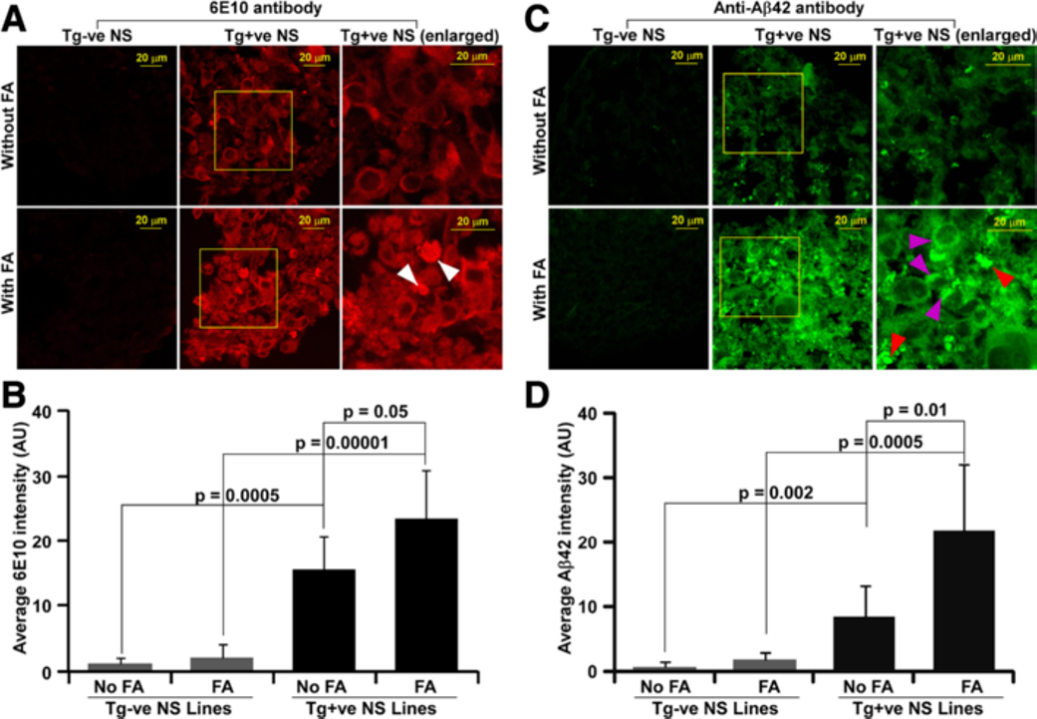
**Detection of Aβ peptides within neurospheres by CDIC. (A)** Neurosphere sections (10 μm thick) were either treated with FA or left untreated, then immunostained with 6E10 antibody. Confocal images were captured using similar exposure settings and displayed with similar intensity scale. White arrowheads indicate the presence of puncta within neurosphere sections. **(B)** Fluorescence intensities were measured and are plotted as histograms of mean + SD of six neurospheres sections. **(C)** Neurosphere sections were also immunostained with an anti-Aβ42 antibody with similar pretreatments. Red and purple arrowheads indicate the presence of puncta outside and within the cells respectively. **(D)** Intensity of Aβ42 fluorescence was measured from six independent neurosphere sections and the mean + SD is plotted as histograms. Student’s *T*-test was performed. p value ≤ 0.05 indicate significant difference between the groups.

Since our neurosphere cultures are much slower growers (split 1 to 3 every 30 to 40 days) than cancerous cell lines and neurospheres under most other culture conditions (split 1 to 4 once a week) (Orr et al. 2012), we speculated Aβ peptides might aggregate within Tg+ve neurospheres. NS sections immunostained with 6E10 antibody and with or without 70% FA treatment showed significant increased immunoreactivity in Tg+ve than Tg-ve NS lines (ANOVA, non parametric, H = 817.827, P < 0.001, n = 6 neurosphere sections) (Figure 11A & B). Moreover, FA treated Tg+ve NS sections, demonstrated significant increased immunoreactivity over untreated counterparts (Figure 11A & B). Immunosignals of puncta were seen within neurosphere sections but outside cellular structures raising the possibility of extracellular aggregation of amyloid-β peptides. In order to address the specificity of 6E10 antibody, NS sections were immunostained with an antibody specific for human Aβ42. Tg+ve NS sections exhibited very high immunostaining in FA treated sections in comparison to untreated Tg+ve NS sections (Figure 11C & D). Small aggregates were seen outside the cellular bodies in FA treated Tg+ve NS sections (Figure 11C, middle and right panel, red arrowheads). In addition, aggregates of Aβ42 immunosignal in the form of puncta are also seen within the cells of FA treated Tg+ve NS sections (Figure 11C, right panel, purple arrowheads). Collectively, our results indicate intracellular and extracellular aggregation of Aβ peptides in Tg+ve neurospheres lines.

## Discussion

In this study, we report a novel way to model the genetics of familial Alzheimer’s disease (FAD) using mouse neurosphere cultures expressing *APPswe* and *PSEN1dE9* mutations of FAD. Using these culture systems we observed the synthesis, secretion, oligomerization and aggregation of human beta amyloid peptides (Aβ40 and Aβ42) better than existing cellular models of AD and comparable to transgenic mouse models of AD (Citron et al. 1997; Oakley et al. 2006).

Several cell culture-based systems from human or rodent, primary or cell lines of neuronal or non-neuronal have been reported. Primary neuronal cultures were preferred over transformed cell lines for two major reasons a) APP expression was thought to be restricted to mature neurons, and b) genomic instability in cancerous cell lines. Although, primary neuronal cultures from transgenic animal models for AD produced both Aβ40 and Aβ42 peptides but survived only for 12 days (Trinchese et al. 2004) or up to 20 weeks (Yun et al. 2007). Recently, induced pluripotent stem cells (iPSCs) derived from fibroblast of FAD patients have been used to model amyloid-β genesis *in vitro* (Yagi et al. 2011). A similar model was developed using fibroblasts from two sporadic and two familial AD patients. Both the FAD lines and one of two lines from patients with sporadic disease show increased Aβ40 levels over wild type cells (Israel et al. 2012). However, the level of Aβ42 is not reported, which increases over Aβ40 in AD patients. In addition, these models utilize transduction of fibroblast with retroviruses encoding *OCT4*, *SOX2*, *KLF4* and *c-MYC*, which are by and large cancerous and form teratomas (Israel et al. 2012). Nevertheless, the advantage of studying patients-derived lines is to determine the influence of varied genetic backgrounds on AD pathology *in vitro*. However, in this report, the role of FAD mutations on mouse neurosphere cultures can be studied on a defined genetic background and these cultures grow continuously for more than 15 passages (at least a year). Thus, our neurosphere cultures offer the advantage of both cell lines and primary cultures.

Unlike iPSC-based culture systems, none of the primary and cancerous cellular model of AD addressed more than one cell type. In this study, neurosphere cultures contain a high percentage of nestin positive CNS stem cells/progenitor cells as reported earlier (Lendahl et al. 1990). Some nestin positive cells express either GFAP (marker for astrocytes) or β-tubulin III (a marker for young and mature neuron) proteins. Cells co-expressing nestin and GFAP has been reported as astroglial progenitor cells (Wei et al. 2002; Draberova et al. 2008). Radial glia cells of human fetal telencephalon and post-natal rat also express GFAP and nestin along with GLAST (Zecevic 2004; Gubert et al. 2009). Thus, nestin and GFAP co-expressing cells in neurosphere cultures suggests the presence of astroglial progenitor and/or radial glia cells. However, we have not tested other markers of radial glial cells such as GLAST and BLBP. Furthermore, co-expression of nestin and neuronal markers, NeuN (Wei et al. 2002) and β-tubulin III (Draberova et al. 2008) has been reported as an indicator of neuronal precursor cells. Therefore, neurosphere cultures in this study contain CNS stem, glial progenitor and neuronal progenitor cells, which may provide additional options to study a variety of differentiated brain cells in the future. Differentiation of our neurosphere cultures produce MAP2^+^ and β-tubulin III^+^ neurons and GFAP^+^ astrocytes without nestin expression (data not shown).

It has been reported that, Aβ peptides are derived from the proteolytic cleavage of APP. Our results from neurosphere lysates (Figure 7) show the expression of full-length humanized APPswe transgene protein and its processed peptides in Tg+ve but not in Tg-ve NS cultures. Presence of C99 peptides suggests the expression of β-secretase in mouse CNS stem cells and its proteolytic activity on transgene-encoded APPswe protein as reported earlier (Vassar et al. 1999). Presence of Aβ peptides also suggests the proteolysis of APP by γ-secretase of which PSEN1 is one of the components. Thus, neurosphere culture system has necessary cellular machineries to produce Aβ peptides from APP.

Aβ peptides are the major insoluble component of the senile plaques in AD and are elevated in AD brain. In AD patients, total Aβ load ranged from 7.8 ng/mg (Wang et al. 1999), 39.824 ng/mg (Ingelsson et al. 2004) to 171 ng/mg (Lue et al. 1999) of total brain proteins. Primary hippocampal cell cultures expressing human *APP* and *PSEN1* mutations produced only 3.28 ng of Aβ peptides from each milligram of total culture supernatant proteins (Trinchese et al. 2004). Purified neurons differentiated from iPSC lines isolated from two FAD patients and one of two sporadic patients secreted only 0.3 ng of Aβ40/mg of lysate (Israel et al. 2012). Transgenic mice expressing five familial Alzheimer’s disease mutations (5XFAD; Tg6799) have been reported to produce Aβ peptides approximately 205 ng/mg and 160 ng/mg of total brain lysate protein in female and male mice respectively at 12 months of age (Oakley et al. 2006). In present study, Tg+ve neurosphere cultures produce monomeric Aβ peptides of 172 ± 96 ng/mg and 128 ± 42 ng/mg of total culture supernatant proteins in NS1 and NS3 neurosphere cultures respectively. Taken together, these neurosphere cultures secrete extracellular Aβ peptides comparable to the concentration of Aβ peptides in human AD brain, 5XFAD mouse brain and much higher level than iPSC and other cellular models of AD.

Aβ40 and Aβ42 are most prevalent Aβ peptides in AD. Tg+ve neurosphere lines secrete both Aβ40 and Aβ42 with Aβ40 amount more than Aβ42. A similar observation has been made in blood plasma and cerebrospinal fluid from AD patients (Bibl et al. 2006) suggesting Aβ40 might be produced more or secreted more efficiently than Aβ42. It has been reported that pathogenic Aβ40 and Aβ42 peptides are produced by neurons, astrocytes (LeBlanc et al. 1997) and oligodendrocytes (Skaper et al. 2009) in AD indicating the expression of APP not only restricted to neurons but also in astrocytes and oligodendrocytes. Furthermore, earlier reports also suggest physiological expression of APP in two-cell embryo, preimplanted and postimplanted embryos, and in the developing neural tube where early neural stem cells are formed (Fisher et al. 1991). Therefore, neural stem/progenitor cells of developing and adult brain might be contributing Aβ peptides to total brain Aβ load in AD patients as well as in the animal model of AD. In continuation to this, mouse prion protein is also expressed in neuron, astrocytes, oligodendrocytes and neural stem cells of adult brain. Therefore, utilization of *Prnp* promoter to express *FAD* genes is a better choice not only for neurons but other brain cells including neural stem/progenitor cells.

Aβ42/Aβ40 ratio has been found to be higher in cells or mice expressing both *APP* and mutant *PSEN1* gene than expressing wild type *APP* alone (Borchelt et al. 1996b; Tomita et al. 1997). In addition, AD patients with *PSEN1/PSEN2*-linked mutations exhibited increased Aβ42/Aβ40 ratio than unaffected individuals and this elevated ratio is considered as a pathological feature of AD (Scheuner et al. 1996). Aβ42/Aβ40 ratio in the culture supernatant of primary hippocampal neurons isolated from mice expressing both human *APP* mutations (K670N: M671L) and human *PSEN1* mutation (M146L) is 0.302 (Trinchese et al. 2004). Differentiated neurons from iPSC-based model of FAD patients harboring a mutation in *PSEN1* (A246E) or *PSEN2* (N141I) genes exhibit Aβ42/Aβ40 ratio as 0.2 (Yagi et al. 2011). Mice expressing *APPswe* and *PSEN1dE9* mutations (similar to present model) exhibited Aβ42/Aβ40 ratio of 1.2 (Jankowsky et al. 2004). Aβ42/Aβ40 ratio in 5XFAD mouse model of AD, have been found to be 1.8 in females and 2.75 in males (Oakley et al. 2006). Such high Aβ42/Aβ40 ratio has been associated with the neurodegeneration seen in this mouse model of AD independent of tauopathy indicating high Aβ42/Aβ40 ratio is an important parameter in modeling beta amyloid pathology of AD *in vitro*. In this study, we demonstrated Aβ42/Aβ40 ratio of 0.875 ± 0.336 and 0.989 ± 0.487 in NS1 and NS3 culture supernatants respectively. To our knowledge, there is no other cellular model of AD available with such a high Aβ42/Aβ40 ratio.

Oligomerization of Aβ peptides has been reported in Tg2576 mouse brain homogenates and their contribution to cognitive deficits independent of plaque load or neuronal loss, suggesting one or more oligomeric forms of Aβ peptides are pathogenic (Cheng et al. 2007; Cleary et al. 2005; Lesne et al. 2006,2013). In this study, we observed oligomers of Aβ peptides ranging from 2-mer to 14-mer in Tg+ve but not in Tg-ve NS lysates and 2-12-mer in Tg+ve culture supernatants. Our results also demonstrate the presence of Aβ-12mer (Aβ*56) peptides in Tg+ve NS culture supernatants. Very recently Aβ trimers, which are thought to be the fundamental amyloid-β assembly unit of Aβ*56, have been reported in young AD mice as well as in 10 years old children (Lesne et al. 2013). In this report we have also observed the presence of both Aβ trimers and Aβ*56 peptides in Tg+ve neurosphere cultures. Therefore, we want to speculate that the pathogenic Aβ production and oligomerization might be happening inside and outside the cells and even in CNS stem or progenitor cells during the early stage of life in AD patients.

Furthermore, presence of Aβ peptides in Tg+ve NS lysates suggest the presence of intracellular Aβ peptides which is well supported by the results from conformation dependent immunocytochemistry on cells and NS sections. Intraneuronal accumulation of Aβ peptide has been associated with cellular pathology related to cognitive malfunction in AD brain (Takahashi et al. 2002; Walsh et al. 2000) and in the brain from mouse models of AD (Eimer and Vassar 2013; Oddo et al. 2006; Wirths et al. 2001; Youmans et al. 2012). Neurotoxic effect (Deshpande et al. 2006) and synaptotoxic effect (Walsh et al. 2002) of oligomeric forms of Aβ have also been reported. Our neurosphere cultures continued to grow and showed no sign of cytotoxicity. This could be due to continuous addition of mitotic factors like EGF, FGF and LIF in the culture medium. Similar to our results, no difference in population doubling was observed between wild type and Tg2576 derived secondary neurospheres, equivalent to passage 1 in this study (Baldassarro et al. 2013).

In addition, the amount of Aβ monomers and oligomers in NS1 is greater than in NS3 culture supernatant in all experiments. We determined that NS1 neurosphere line is female and NS3 is male (for genotyping result, see Additional file 2: Figure S2). It has been shown that female *APP/PSEN1* mice build up more amyloid deposits than age matched male mice (Wang et al. 2003; Sierksma et al. 2013). Epidemiological studies also suggest an increased risk for AD among females compared to age-matched males (Andersen et al. 1999). Collectively, our neurosphere culture manifested higher Aβ load, high Aβ42/Aβ40 ratio and gender bias towards Aβ synthesis and oligomerization, which parallels the situation in AD patients and in animal models that express *APPswe* and *PSEN1dE9* mutations.

The effect of Aβ peptides on neural progenitors cells (NPC) is controversial. Proliferation and survival of NPC in the dentate gyrus of the hippocampus was reduced in mice transgenic for FAD mutant *APP*. Reduced neuronal cell survival was seen after Aβ treatment of cultured human and rodent NPC (Haughey et al. 2002). Uchida et al. have also reported acceleration of differentiation followed by death in Aβ treated neural stem/progenitor cells (Uchida et al. 2000). In contrast to these reports, increased hippocampal neurogenesis in AD patients and Aβ treated neural stem cell cultures from striatum and hippocampus have been reported (Jin et al. 2004; Lopez-Toledano and Shelanski 2004). Most of these reports employed exogenous Aβ peptides treatments. However, our neurosphere cultures secrete Aβ peptides endogenously, ranging from monomeric to various pathogenic oligomeric forms. Whether CNS stem/progenitor cells-enriched neurosphere cultures are affected by being bathed in Aβ peptides is not known. Our future studies will address such questions using these Aβ peptide producing NS cultures.

## Conclusion

Our results for the first time report that CNS stem/progenitor cell-enriched neurosphere cultures express majorities of the Aβ peptide pathologies that are seen in AD patients and animal models of AD. The advantages our neurosphere cultures offer over existing cellular models are 1) these cultures offer both primary and cancerous cell benefits, and contain CNS stem/progenitor like cells, which can differentiate towards mature brain cells like neurons and astrocytes that are not possible in transformed cell lines, 2) synthesize and secrete both Aβ peptides, 3) demonstrate high Aβ42/Aβ40 ratio, 4) produce pathogenic Aβ peptide oligomerization (including toxic Aβ 12-mer; Aβ*56 peptide) at a level comparable to the animal models of AD and much higher than existing cellular models of AD, including iPSC based models of AD and 5) demonstrate intracellular and extracellular aggregation of Aβ peptides. Having such strong Aβ peptide pathologies, differentiated cells from these cultures in future will advance our understanding on cellular and molecular changes in response to endogenously produced Aβ peptides and possible therapeutic studies to decrease beta amyloid synthesis and aggregation within cells.

## Electronic supplementary material

Additional file 1: Figure S1: Effect of heat induced epitope retrieval (HIER) on APP, GAPDH and Aβ peptides. (A) Sixty μg of total protein from a Tg+ve and Tg-ve mouse brain homogenate was size fractionated in 16% Tris-Glycine-SDS-PAGE along with 25 ng of Aβ42 monomeric peptides. Proteins were transferred onto 0.2 μm nitrocellulose membrane and immunoblotted with 6E10 antibody with or without HIER. Digital images were captured using a ChemiDoc XRS^+^gel doc system (BIO-RAD, USA). Images from blots with or without HIER treatment are displayed with identical image intensity scale. After the completion of imaging, the blots were stripped and immunoblotted with an antibody specific for GAPDH. (B) Densitometric analysis of bands corresponding to APP (full length), Aβ monomers and GAPDH were made using ImageLab (version 3.0) software. Results indicate HIER increased the APP, GAPDH and Aβ42 signal by 5.57, 10.38 and 120.39 fold respectively than without HIER suggesting Aβ peptides require HIER treatment approximately 21-fold more than APP and 12-fold more than GAPDH. HMW = High molecular weight, FL = full length. (PDF 681 KB)

Additional file 2: Figure S2: Detection of male specific gene, *SRY* by polymerase chain reaction (PCR). Genomic DNA was isolated from neurosphere cultures and also from a male mouse. A DNA PCR was used to amplify SRY gene (specific for maleness in mouse) using forward primer as 5′-AGGCACAAGTTGGCCCAGCA-3′ and reverse primer as 5′-TGTGGGTTCCTGTCCCACTGCA-3′. Result indicates a band of 269 bp was amplified from the genomic DNA of NS3 and a male mouse but not from NS2, NS4 and NS1. Thus, NS3 neurosphere is a male neurosphere whereas NS1, NS2 and NS4 are female neurospheres. Mo = Mouse. (PDF 254 KB)

## Abbreviations

AD: Alzheimer’s disease
FAD: Familial Alzheimer’s disease
APP: Amyloid precursor protein
PSEN: Presenilin
Aβ: Amyloid beta
CNS: Central nervous system
NPC: Neural progenitors cells
MBH: Mouse brain homogenate
CDIC: Conformation dependent immunocytochemistry
HIER: Heat induced epitope retrieval
FA: Formic acid
NS: Neurosphere
Tg: Transgenic.
