# Supplementary figures and images for: Development of a novel cellular model of Alzheimer’s disease utilizing neurosphere cultures derived from B6C3-Tg(APPswe,PSEN1dE9)85Dbo/J embryonic mouse brain
Source: Springerplus. 2014 Mar 26;3:161. doi: 10.1186/2193-1801-3-161 (PMC4137416; doi:10.1186/2193-1801-3-161)

**A**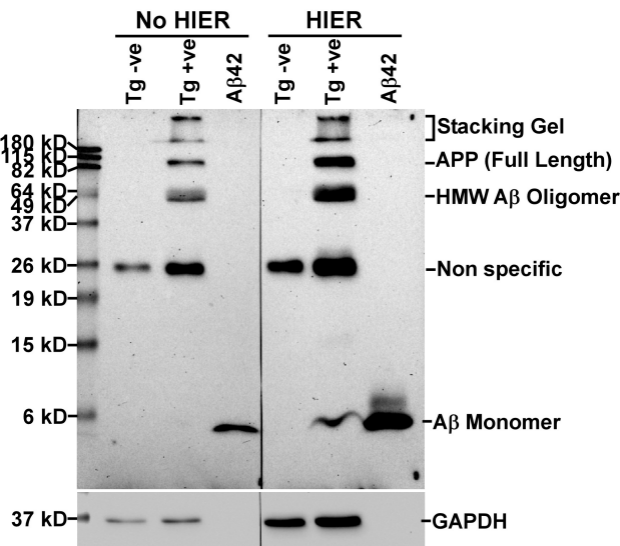**B**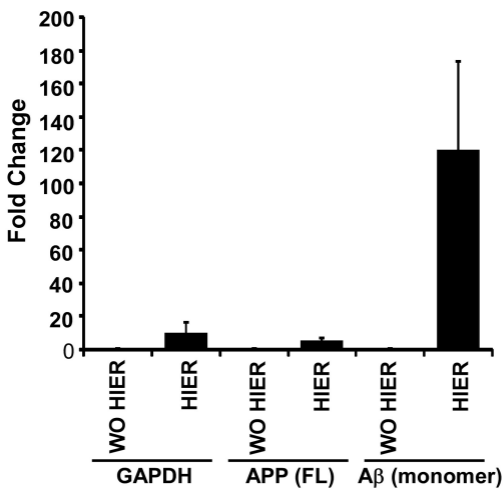

**Supplementary Figure 1**

Supplement: Supplementary file 1 — Additional file 1: Figure S1: Effect of heat induced epitope retrieval (HIER) on APP, GAPDH and Aβ peptides. (A) Sixty μg of total protein from a Tg+ve and Tg-ve mouse brain homogenate was size fractionated in 16% Tris-Glycine-SDS-PAGE along with 25 ng of Aβ42 monomeric peptides. Proteins were transferred onto 0.2 μm nitrocellulose membrane and immunoblotted with 6E10 antibody with or without HIER. Digital images were captured using a ChemiDoc XRS+gel doc system (BIO-RAD, USA). Images from blots with or without HIER treatment are displayed with identical image intensity scale. After the completion of imaging, the blots were stripped and immunoblotted with an antibody specific for GAPDH. (B) Densitometric analysis of bands corresponding to APP (full length), Aβ monomers and GAPDH were made using ImageLab (version 3.0) software. Results indicate HIER increased the APP, GAPDH and Aβ42 signal by 5.57, 10.38 and 120.39 fold respectively than without HIER suggesting Aβ peptides require HIER treatment approximately 21-fold more than APP and 12-fold more than GAPDH. HMW = High molecular weight, FL = full length. (PDF 681 KB) [file 40064_2013_1127_MOESM1_ESM.pdf]

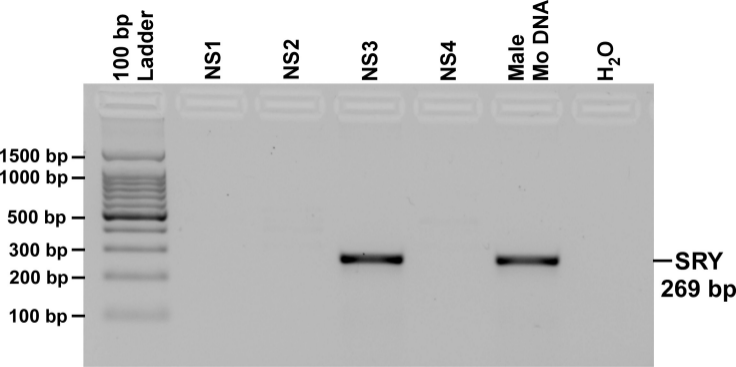

**Supplementary Figure 2**

Supplement: Supplementary file 2 — Additional file 2: Figure S2: Detection of male specific gene, SRY by polymerase chain reaction (PCR). Genomic DNA was isolated from neurosphere cultures and also from a male mouse. A DNA PCR was used to amplify SRY gene (specific for maleness in mouse) using forward primer as 5′-AGGCACAAGTTGGCCCAGCA-3′ and reverse primer as 5′-TGTGGGTTCCTGTCCCACTGCA-3′. Result indicates a band of 269 bp was amplified from the genomic DNA of NS3 and a male mouse but not from NS2, NS4 and NS1. Thus, NS3 neurosphere is a male neurosphere whereas NS1, NS2 and NS4 are female neurospheres. Mo = Mouse. (PDF 254 KB) [file 40064_2013_1127_MOESM2_ESM.pdf]
